# Functional and Structural Characterization of Purine Nucleoside Phosphorylase from *Kluyveromyces lactis* and Its Potential Applications in Reducing Purine Content in Food

**DOI:** 10.1371/journal.pone.0164279

**Published:** 2016-10-21

**Authors:** Durga Mahor, Anu Priyanka, Gandham S Prasad, Krishan Gopal Thakur

**Affiliations:** 1 Microbial Type Culture Collection and Gene Bank, CSIR-Institute of Microbial Technology, Chandigarh, India; 2 Structural Biology Laboratory, G. N. Ramachandran Protein Centre, CSIR-Institute of Microbial Technology, Chandigarh, India; Leibniz-Institut fur Pflanzengenetik und Kulturpflanzenforschung Gatersleben, GERMANY

## Abstract

Consumption of foods and beverages with high purine content increases the risk of hyperuricemia, which causes gout and can lead to cardiovascular, renal, and other metabolic disorders. As patients often find dietary restrictions challenging, enzymatically lowering purine content in popular foods and beverages offers a safe and attractive strategy to control hyperuricemia. Here, we report structurally and functionally characterized purine nucleoside phosphorylase (PNP) from *Kluyveromyces lactis* (*Klac*PNP), a key enzyme involved in the purine degradation pathway. We report a 1.97 Å resolution crystal structure of homotrimeric *Klac*PNP with an intrinsically bound hypoxanthine in the active site. *Klac*PNP belongs to the nucleoside phosphorylase-I (NP-I) family, and it specifically utilizes 6-oxopurine substrates in the following order: inosine > guanosine > xanthosine, but is inactive towards adenosine. To engineer enzymes with broad substrate specificity, we created two point variants, *Klac*PNP^N256D^ and *Klac*PNP^N256E^, by replacing the catalytically active Asn256 with Asp and Glu, respectively, based on structural and comparative sequence analysis. *Klac*PNP^N256D^ not only displayed broad substrate specificity by utilizing both 6-oxopurines and 6-aminopurines in the order adenosine > inosine > xanthosine > guanosine, but also displayed reversal of substrate specificity. In contrast, *Klac*PNP^N256E^ was highly specific to inosine and could not utilize other tested substrates. Beer consumption is associated with increased risk of developing gout, owing to its high purine content. Here, we demonstrate that *Klac*PNP and *Klac*PNP^N256D^ could be used to catalyze a key reaction involved in lowering beer purine content. Biochemical properties of these enzymes such as activity across a wide pH range, optimum activity at about 25°C, and stability for months at about 8°C, make them suitable candidates for food and beverage industries. Since *Klac*PNP^N256D^ has broad substrate specificity, a combination of engineered *Klac*PNP and other enzymes involved in purine degradation could effectively lower the purine content in foods and beverages.

## Introduction

Cellular and dietary nucleotides and nucleic acids are continuously degraded in living cells, where purine and pyrimidine degradation pathways play an important role. The purine degradation pathway involves an oxidative purine ring cleavage, forming uric acid as a final product in humans. Uric acid has poor solubility in biological fluids and results in hyperuricemia at elevated levels, which increases the risk of developing gout [1, 2]. Gout is the most common inflammatory arthritis, characterized by deposition of uric acid crystals, predominantly at the joints [3, 4]. Hyperuricemia is also an important risk factor for cardiovascular, renal, and metabolic diseases [5–9]. Studies suggest that besides several non-modifiable risk factors like genetics, race, sex, and age, some modifiable risk factors such as diet and lifestyle are also associated with gout [10]. Increased prevalence of hyperuricemia, use of low-dose aspirin, occurrence of obesity and metabolic syndrome, alcohol consumption, longevity, rampant use of diuretics, and dietary trends are all contributing factors to the increased prevalence of gout, especially in developed countries [11–19]. There are various uricostatic, uricosuric, and uricolytic drugs [20–25] that cure gout, depending on the clinical stage of the disease. These medical interventions are associated with some drawbacks: antigenicity, high cost, and other undesirable side-effects [26–29]. Besides medication, alternative approaches like consuming foods and beverages with low purine and sugar content, and changing lifestyle can help control the uric acid level in the body [30, 31]. There are reports suggesting that among the alcoholic drinks, beer consumption increases the risk of gout [32, 33]. Hence, limiting the consumption of beer is advisable for patients suffering from gout [34–36].

**Fig 1:**
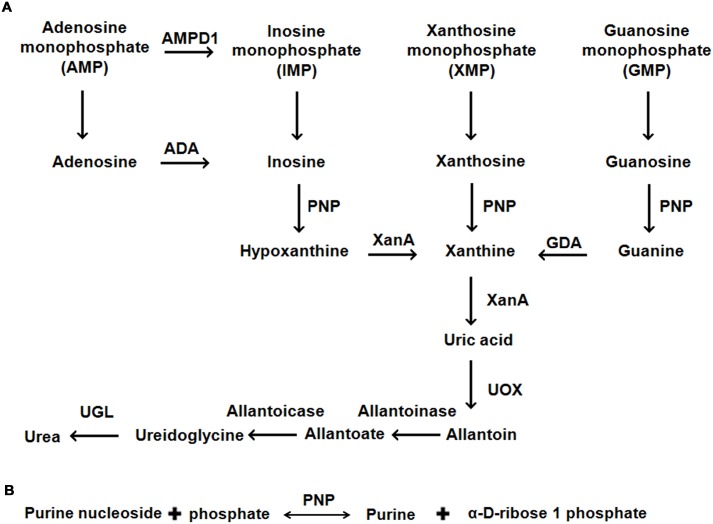
Purine degradation pathway in *K*. *lactis*. (A) Schematic representation of the purine degradation pathway in *K*. *lactis* (KEGG pathway). (B) Enzymatic reaction carried out by PNP. The abbreviations used are: AMPD1, Adenosine monophosphate deaminase 1; ADA, Adenosine deaminase; PNP, Purine nucleoside phosphorylase; XanA, Xanthine dioxygenase; GDA, Guanine deaminase; UOX, Urate oxidase; and UGL, ureidoglycolate lyase.

Since changing dietary habits is difficult [37], an alternative way to reduce dietary intake of purines is to produce foods and beverages with low purine content, using enzymatic degradation [38]. Characterization of purine degrading enzymes is essential to apply this approach. PNP, one of the key enzymes involved in the purine degradation pathway is well studied in bacteria and mammals [39–42]. However, there are limited biochemical studies of the enzyme [43, 44], and no structural information is currently available for the yeast homologs. The yeast, *Kluyveromyces lactis*, is generally recognized as safe (GRAS) as per FDA status and our group is working on structural and functional characterization of the enzymes involved in its purine degradation pathway. The purine degradation pathway of *K*. *lactis* includes the following enzymes: adenosine deaminase (ADA), guanine deaminase (GDA), purine nucleoside phosphorylase (PNP), xanthine dioxygenase (XanA), urate oxidase (uricase), allantoinase, allantoicase, and ureidoglycolate lyase (UGL) (Fig 1A).

PNP (PNP: E.C. 2.4.2.1) catalyzes the reversible phosphorolysis of N-ribosidic bonds of nucleosides and generates a purine base and ribose-1- phosphate (Fig 1B). On the basis of its oligomeric state, molecular weight, and substrate specificity, PNP has been classified into two different classes. The homotrimeric class enzymes utilize 6-oxopurines as substrates, while the homohexameric class enzymes utilize 6-oxopurines and 6-aminopurines. Previous studies in homotrimeric human PNP revealed that the active site-binding residue, Asn243, plays an important role in catalysis and is responsible for substrate specificity as well [45–48]. In contrast, in the active site of hexameric PNPs, with few exceptions, this structurally equivalent residue is replaced by Asp residue, which broadens their substrate specificity [49]. Therefore, to engineer enzymes with broad substrate specificity, we replaced the structurally equivalent Asn256 residue to generate two variants: *Klac*PNP^N256D^ and *Klac*PNP^N256E^. In the present study, we report the structural and biochemical characterization of *K*. *lactis* PNP (*Klac*PNP). We report the crystal structure of *Klac*PNP at 1.97 Å resolution and observed an extra electron density at the active site. Using structural and mass spectroscopy studies, we could identify this intrinsically bound ligand. Our biochemical data suggest that *Klac*PNP could not metabolize 6-aminopurines. However, to render the process of lowering purine content by using an enzymatic approach cost effective it is desirable to minimize the number of enzymes used in the process. Hence, using sequence and structural information, we identified a key residue that dictates substrate specificity and created two point variants to broaden substrate specificity in *Klac*PNP, as described above. We present the biochemical characterization of *Klac*PNP and its point variants. We also demonstrate the potential applications of *Klac*PNP and its point variant in catalyzing one of the key reactions involved in lowering purine content in a beer sample. We also propose possible future strategies that can be utilized for the production of foods and beverages with low purine content.

## Materials and Methods

### Cloning, overexpression, and purification of *Klac*PNP, *Klac*PNP^N256D^, and *Klac*PNP^N256E^ enzymes

Genomic DNA of *K*. *lactis* (MTCC 458 CSIR-IMTECH, India) was isolated by using ZR Fungal/Bacterial DNA MicroPrep kit from Zymo Research, USA. Amplified *pnp* gene product was cloned between NdeI/HindIII and NdeI/NotI restriction sites into pET28c and pET28a-BsaI to yield N-terminal and C-terminal 6xHis-tags, respectively. *Klac*PNP^N256D^ and *Klac*PNP^N256E^ mutants were created using a PCR-based site-directed mutagenesis protocol. All clones were confirmed by DNA sequencing. The recombinant colonies harboring wild type or mutant *pnp* plasmids were inoculated in 10 mL LB medium containing 50 μg/mL kanamycin and incubated overnight at 37°C with constant shaking at 150 rpm. Primary culture (10 mL) was further inoculated in 1 L LB medium for large-scale production. Cells were harvested by centrifugation at 5612 × *g* for 15 min at 4°C. The pellet was re-suspended in lysis buffer (50 mM Tris-HCl, 200 mM NaCl, 10 mM imidazole, pH 8.0) and incubated for 30 min with lysozyme (1 mg/mL) and protease cocktail inhibitor (1 μL/mL, Sigma-Aldrich). Cells were disrupted by sonication, and the cell debris was separated by centrifugation at 18400 × *g* at 4°C for 45 min. The supernatant was loaded onto the Ni-NTA column that was pre-equilibrated with equilibration buffer (50 mM Tris-HCl, 200 mM NaCl, 10 mM imidazole, pH 8.0). The bound protein was eluted from the Ni-NTA column by using elution buffer (50 mM Tris-HCl, 200 mM NaCl, 200 mM imidazole, 10% glycerol, pH 8.0) in several fractions. Elution fractions were pooled and dialyzed overnight against buffer (25 mM Tris-HCl, 100 mM NaCl, 1 mM DTT, pH 8.0). The dialyzed recombinant protein was concentrated by using an Amicon ultrafiltration device (Merck Millipore) and further purified by gel filtration chromatography by using HiPrep 16/60 Sephacryl S-200 HR column (GE Healthcare Life Sciences). The purity and molecular mass of *Klac*PNP were determined by SDS-PAGE and MALDI-TOF mass spectrometry (Voyager DESTR mass spectrometer, Applied Biosystems), respectively.

### Protein crystallization, data collection, structure determination, and refinement

Several crystallization trials were set up by using commercial screens from Hampton Research, using 96-well vapor diffusion sitting drop trays. Both N-terminal and C-terminal 6xHis-tag protein variants were screened for crystallization. Only C-terminal 6xHis-tag variant protein showed good diffraction quality crystals. Data were collected from the crystals grown by mixing 1.25 μL of 8 mg/mL of protein in 25 mM Tris-HCl, 100 mM NaCl, pH 8.0 mixed with 1.25 μL of mother liquor, consisting of 0.1 M sodium acetate trihydrate, pH 4.6, and 8% PEG4000. Data were collected on beamline BM14 at ESRF, Grenoble, France. Data were processed by using iMOSFLM [50] and scaled by using SCALA [51] to a resolution of 1.97 Å. PHASER [52] was used to solve the structure by molecular replacement, using human PNP as a search template (PDB ID 3PHB) [53]. Several iterative rounds of manual model building by using COOT [54] and refinement by using PHENIX.REFINE [55] were used to generate a model with R and R_free_ of 0.15 and 0.20, respectively. The model has excellent geometric parameters with 99.08% of residues lying in the allowed region and 0.92% in the generously allowed region. The final data collection and refinement statistics are provided in Table 1. All molecular graphics were drawn by using Pymol [56].

**Table 1:** Data collection and refinement statistics.

*Klac*PNP	
**Data Collection**	
Space Group	P 6_1_
Cell dimensions	
a, b, c (Å)	80.78, 80.78, 231.16
α, β, γ (°)	90, 90, 120
Resolution (Å)	39.79–1.97 (2.07–1.97)*
R_merge_	0.059 (0.34)
*I*/σ*I*	16.0 (4.4)
Completeness (%)	99.7 (98.6)
Redundancy	4.8 (4.6)
Wavelength (Å)	0.97625
**Refinement**	
Resolution (Å)	39.79–1.97
No. of unique reflections	60087 (8665)
R_work_ /R_free_	0.156/0.199
Residue/atom count	
Protein	7438
Water	626
Ligand	30
B- factor	
Protein	34.7
Water	39.5
Ligand	26.6
r.m.s.d.^#^	
Bond lengths (Å)	0.0076
Bond angles (°)	1.075
**Structure Validation**	
Ramachandran plot statistics	
Most favored (%)	99.08%
Allowed regions (%)	0.92%
Disallowed region (%)	0.0%

* Values in the parentheses correspond to the highest resolution shells.

^#^ r.m.s.d., root mean squared deviation.

### Identification of intrinsically bound ligand by electrospray ionization mass spectrometry (ESI-MS) analysis

To identify the intrinsically bound ligand, *Klac*PNP was heat denatured to release the bound ligand. Briefly, 0.5 mL of about 75 μM *Klac*PNP in buffer having 10 mM Tris, 20 mM NaCl, pH 8.0, was incubated at 95°C for 30 min. The aggregated protein was pelleted by spinning at 14549 × *g* for 30 min. The supernatant containing the released ligand was subjected to direct injection ESI-MS analysis in an LC-MS system (model G6550A, Agilent Technologies). The sample was injected at a flow rate of 0.05 mL/min and resolved in 50% chloroform and 50% acetonitrile solvent system. Mass range was scanned between 100 Da to 200 Da with a scan rate of 2 spectra/s.

### Enzymatic assay of *Klac*PNP and its point variants, *Klac*PNP^N256D^ and *Klac*PNP^N256E^

The PNP activity was measured by using a coupled reaction with xanthine oxidase. Hypoxanthine produced from the degradation of inosine by PNP is followed by its two-step transformation into uric acid, using commercial xanthine oxidase (0.1 U/reaction). The production of uric acid in the reaction was monitored by measuring its specific absorbance at 293 nm [43, 57]. The reactions were performed in 100 mM potassium phosphate buffer (pH 7.0) at 25°C. The kinetic parameters of *Klac*PNP, *Klac*PNP^N256D^, and *Klac*PNP^N256E^ enzymes were determined for inosine, guanosine, xanthosine, and adenosine as starting substrates. Substrate concentrations varied between 0 and 5 mM. The reactions were stopped at 10, 15, 20, and 30 min, so that measurements remained in the linear range. Kinetic parameters were determined and plotted by using a non-linear curve fit module in Origin software.

### Biochemical properties of *Klac*PNP and its point variants, *Klac*PNP^N256D^ and *Klac*PNP^N256E^

Optimum pH conditions for *Klac*PNP, *Klac*PNP^N256D^, and *Klac*PNP^N256E^ enzymes were determined by examining the activity in a pH range from 3 to 12, at an interval of 1 pH unit, in 100 mM universal buffer (50 mM Tris, 50 mM boric acid, 33 mM citric acid, and 50 mM Na_2_PO_4_) [58] adjusted with either HCl or NaOH to obtain the desired pH. The reaction mixture (250 μL) was incubated at 25°C for 20 min, and the reaction was stopped by adding 50 μL of 10 M NaOH. Enzyme activity was measured at 293 nm in a microplate reader. The effect of temperature on the activity of enzymes was examined by incubating the reaction mix at different temperatures at their optimum pH. Different incubation temperatures ranging from 5°C to 55°C (5°C intervals) were used. The assay mixture was pre-incubated at different temperatures. The enzyme was added to the reaction mixture and incubated at the appropriate temperature for 20 min. The reaction was stopped by the addition of 50 μL of 10 M NaOH solution and readings were taken immediately.

### Temperature and pH stability of *Klac*PNP, *Klac*PNP^N256D^, and *Klac*PNP^N256E^ enzymes

*Klac*PNP and its mutant variants were incubated at 25°C for 12 h and 24 h in the universal buffer, which was set at different pH values, as mentioned above. Post incubation, the enzyme assay was performed at the optimum conditions obtained from the previous experiments. The reaction was stopped by addition of 50 μL of 10 M NaOH, and the absorbance was measured at 293 nm. To characterize temperature stability, *Klac*PNP, *Klac*PNP^N256D^, and *Klac*PNP^N256E^ in buffer with at pH 7.0, 6.0, and 7.0, respectively, were incubated at different temperatures from 5°C to 55°C for 2 h. Next, the enzyme was added to the reaction mixture and absorbance was measured at 293 nm.

### Nucleoside degradation analysis in a beer sample

An HPLC-based method was optimized to detect the nucleosides and nucleotides in the standards and beer sample. Nucleosides and nucleotides (inosine, guanosine, xanthosine, adenosine, hypoxanthine, guanine, xanthine, and adenine) at a concentration of 2 mM were dissolved in 100 mM phosphate buffer (pH 7.0). All the samples were filtered through 0.22 μm syringe filters. Ten microliters of sample was injected in a Lichrospher 100 RP-18 column (LichroCART ^®^250–4, Merck), and UV-absorbance spectra were collected at 254 nm. HPLC analyses were performed at 35°C with 0.5 mL/min flow rate on an SCL-10AV HPLC system (Shimadzu, Japan), equipped with an SIL-10AD injector and a UV spectrophotometric detector. The mobile phase was as follows: 100 mM sodium phosphate buffer, 10% methanol, pH 2.3. The desired pH of sodium phosphate buffer was adjusted with 100 mM phosphoric acid. To evaluate the purine degradation, each nucleoside (substrate) was treated with *Klac*PNP (0.8 U) at 25°C for 20 min. The reaction mixture was then heated at 100°C for 5 min, centrifuged, and loaded onto the HPLC system. We could not detect a significant amount of adenosine in the beer sample that we tested. Therefore, to analyze adenosine degradation in the presence of *Klac*PNP^N256D^, we added adenosine at a final concentration of 20 mg/L to the beer sample. The beer sample was treated by adding 0.8 U of *Klac*PNP and *Klac*PNP^N256D^, incubated at 25°C for 20 min. HPLC experiments were performed by using the above-mentioned parameters. Chromatograms of samples obtained in the absence and presence of enzyme were compared to evaluate the potential of *Klac*PNP and its mutant variant *Klac*PNP^N256D^ in reducing the purine content in the beer sample. The identity of the compounds was assigned based on the retention time of the standards in the chromatogram, and further confirmed by using LC-ESI-MS analysis.

## Results

### Cloning, expression, and purification of *Klac*PNP, *Klac*PNP^N256D^, and *Klac*PNP^N256E^

**Fig 2:**
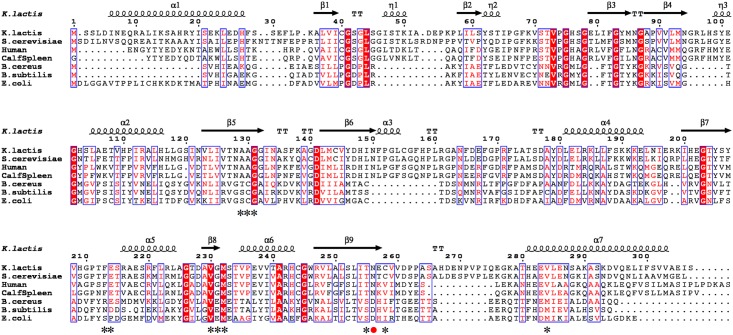
Multiple sequence alignment of *Klac*PNP homologs. Sequence comparison of *Klac*PNP with homotrimeric PNPs (specific for 6-oxopurines: *S*. *cerevisiae*, human, and calf spleen PNPs), homohexameric PNPs (specific for 6-aminopurines: *B*. *cereus*, *B*. *subtilis*, and *E*. *coli* PNPs). Highly conserved regions are highlighted with red boxes; conservative substitutions are also boxed. The figure was drawn by using the ESPript 3 server [59]. Active site residues involved in the interaction with hypoxanthine are shown with an asterisk and the catalytically active residue that is known to play an important role in substrate specificity is shown with a red filled circle [45–49].

A 921 bp sequence containing the complete *K*. *lactis pnp* gene was retrieved from GenBank (Accession number: XM 452943.1). Sequence analysis revealed that *Klac*PNP shares sequence homology with *S*. *cerevisiae* PNP (66%), human PNP (47%), calf spleen PNP (47%), *Escherichia coli* PNP (29%), *Bacillus cereus* PNP (27%), and *Bacillus subtilis* PNP (27%) (Fig 2).

The presence of a catalytically active Asn or Asp residue at the structurally equivalent position in trimeric and hexameric PNPs, respectively, is known to play an important role in substrate specificity [45–49] (Fig 2). On the basis of multiple sequence alignment and structural analysis, we identified the residue dictating substrate specificity, and created *Klac*PNP^N256D^ and *Klac*PNP^N256E^ point variants (Fig 2). *Klac*PNP, *Klac*PNP^N256D^, and *Klac*PNP^N256E^ were overexpressed and purified by Ni-NTA chromatography. Fractions containing purified *Klac*PNP, *Klac*PNP^N256D^, and *Klac*PNP^N256E^ were pooled, concentrated, and further purified by size exclusion chromatography. The protein could be purified to homogeneity with >95% purity, as inferred from SDS-PAGE analysis. The protein could be concentrated to >20 mg/mL. *Klac*PNP was stable for months when stored at 8°C in an elution buffer (25 mM Tris-HCl, 100 mM NaCl, 10% glycerol, pH 7.0). Up to 65% activity was retained after storage in the above-mentioned conditions for over 30 days. Protein concentration estimated by using UV-absorbance was 20% higher than that estimated by using the Bradford method. This discrepancy was later resolved, as the crystal structure revealed that hypoxanthine was bound to the active site, which contributed to the overestimation of protein concentration.

### *Klac*PNP exists as a homotrimer in solution

**Fig 3:**
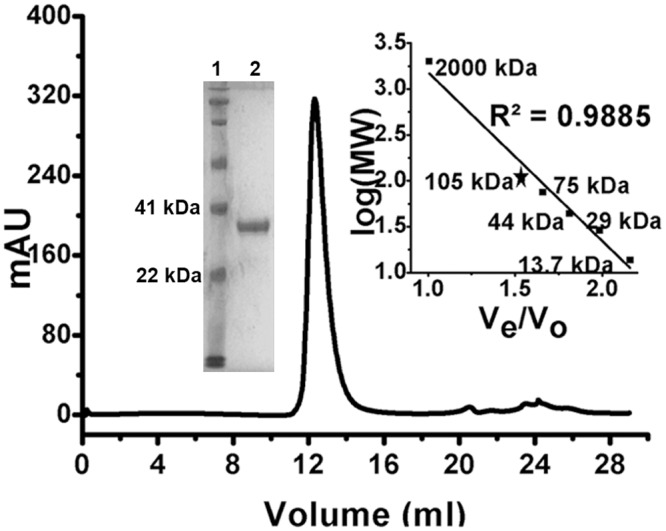
Analytical gel filtration profile of *Klac*PNP. Analytical size exclusion chromatography elution profile of *Klac*PNP suggests a homotrimeric oligomeric state of *Klac*PNP. The inset graph shows the elution profile of standard molecular weight marker proteins: ribonuclease (13.7 kDa), carbonic anhydrase (29 kDa), ovalbumin (44 kDa), conalbumin (75 kDa), PNP (star-marked: about 105 kDa), and blue dextran (about 2000 kDa).

PNP proteins fall into two distinct classes based on their oligomeric state: homotrimeric and homohexameric. PNPs of mammalian origin reported so far are homotrimeric, and most PNPs of bacterial origin are homohexameric [41, 60]. Since there is no report on the oligomeric state of yeast PNPs, we performed analytical size exclusion chromatography to study the oligomeric state of *Klac*PNP. The protein eluted as a single predominant peak at 12.5 mL on a Superdex 200 Increase 10/300 GL analytical gel filtration column, corresponding to an apparent molecular weight of 105 kDa, suggesting a homotrimeric state of *Klac*PNP (Fig 3). Based on the observed oligomeric state in solution, *Klac*PNP can be classified in the low molecular mass, homotrimeric class of NP-I family of nucleoside phosphorylases [60].

### Overall structure of *Klac*PNP

**Fig 4:**
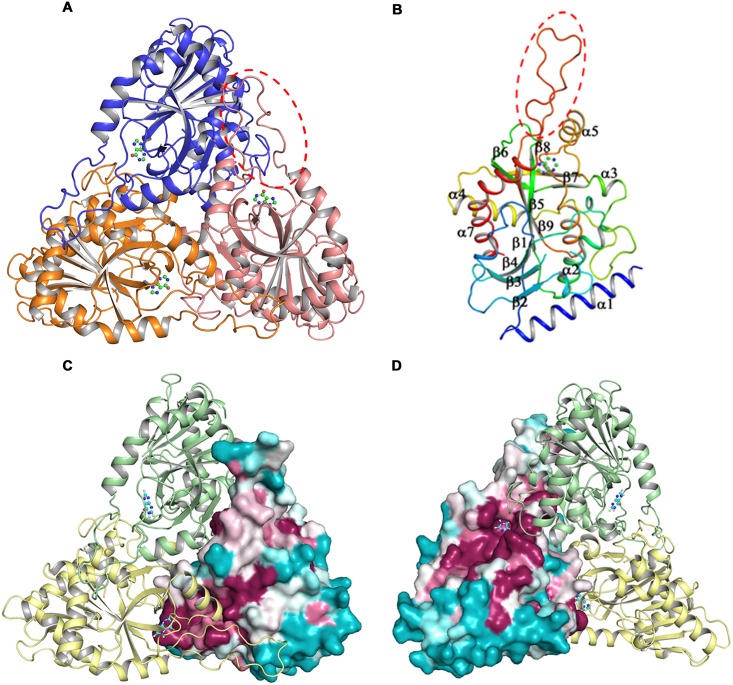
Crystal structure of *Klac*PNP. (A) The structure reveals the trimeric arrangement of *Klac*PNP. Hypoxanthine bound to the active site is shown as a ball and stick representation. (B) Monomer showing arrangement of secondary structure elements colored in rainbow format. The long loop region connecting α7 and β9, which forms extensive direct contacts between the monomers forming the trimer, can be observed in red. (C and D) Consurf [63] analysis showing high conservation scores for residues present in the core, active site, and at the intersubunit interface.

**Fig 5:**
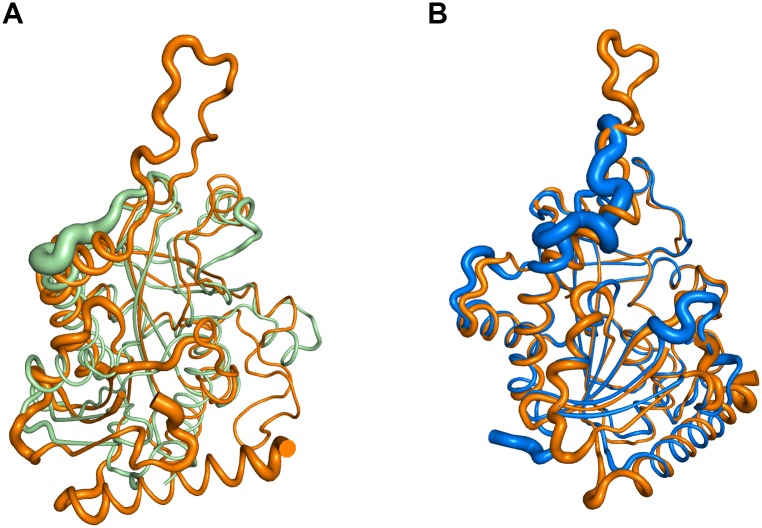
Structural similarity between *Klac*PNP with human and *E*. *coli* PNP. (A and B) Structural superimpositions in putty representation showing the structural homology of *Klac*PNP monomer with human PNP and *E*. *coli* PNP. The thick regions in the putty, localized primarily at high B-factor loop regions, have higher structural deviations. The large differences in the loop regions and presence of an extra helix at the N-terminal region of *Klac*PNP can be observed in the panel.

*Klac*PNP crystallized in the space group P6_1_. There were three molecules in the asymmetric unit, corresponding to one biological trimer of *Klac*PNP (Fig 4A). All three chains superimposed well with an RMSD of less than 0.3 Å. Four residues at the N-terminus, and a stretch of seven residues connecting β2 and β3 between Val70 and Gly78, could not be modeled because of disorder or high B-factor. All three monomers assembled to form a trimer having a non-crystallographic, 3-fold symmetry. This is in agreement with the in solution oligomeric state of *Klac*PNP determined by the analytical gel filtration experiment. The overall structure of *Klac*PNP is similar to those of other reported mammalian PNPs i.e., human and calf PNPs [48, 61]. A trimer is formed from the A-B, B-C, and A-C interfaces. Each interface buries approximately 1550 Å^2^ and is stabilized by on an average 24 hydrogen bonds, 4 salt bridges, and several non-bonded interactions. Residues from 3_10_3, α3, loops connecting β7/α5 and β9/α7 of one monomer are involved in the interactions with β6, α3, and β7 of the other monomer. The longest stretch of residues involved in these intermolecular interactions is formed by a long loop connecting β9 and α7. Interestingly, this loop region is the longest among all the structurally characterized PNPs to date (Fig 4B). Thr234, which forms a part of the tight gamma turn and is involved in phosphate binding, is structurally equivalent to Thr221 of the calf spleen PNP [61], which is reported to lie in the unfavorable region in the Ramachandran plot. Though structural validation performed by using COOT [54] showed the presence of *Klac*PNP Thr234 in the disallowed region, validation by using Molprobity [62] showed no residue in the disallowed region of the Ramachandran Plot. There are three cysteine residues in the sequence, and none of them is involved in the formation of a disulfide bond. Interestingly, Cys155 in α3 from each monomer is located close to the 3-fold non-crystallographic symmetry axis, and all three SH groups facing towards the core lie within 3.5 Å distance, well within a range to form a disulfide bond under suitable conditions. However, we did not observe a disulfide bond. Instead, a water molecule forms a tripartite hydrogen bond with the three free SH groups. To identify structurally conserved residues, we performed Consurf analysis [63]. Besides showing high conservation scores for the core and the active site residues, Consurf analysis also revealed high conservation scores for the residues lining the intersubunit interface (Fig 4C and 4D). Structural superposition of the *Klac*PNP monomer over human and *E*. *coli* homologs revealed that the core of the protein had lower RMSD whereas, as expected, the loop regions showed higher RMSD. In addition to the long loop region, another distinguishing feature of *Klac*PNP is the presence of an N-terminal helix, α1. (Fig 5A and 5B).

**Fig 6:**
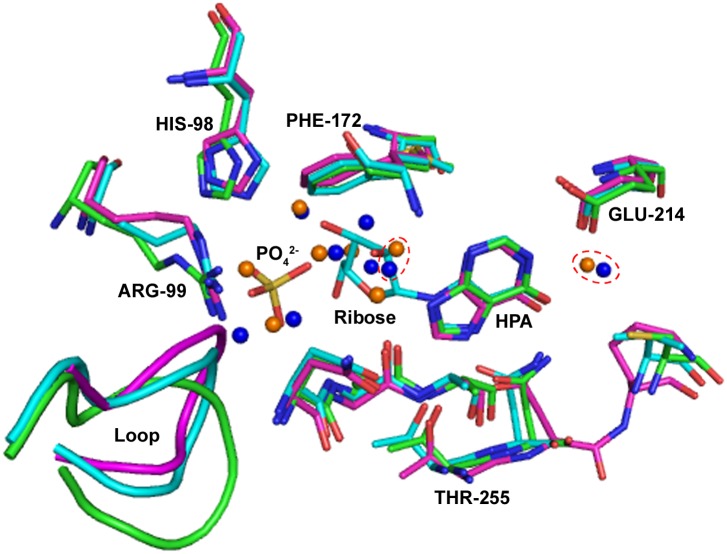
Comparative structural analysis of the active sites of PNPs. Crystal structures of *K*. *lactis* (green, hypoxanthine bound structure), calf spleen (magenta, PDB ID 1VFN, hypoxanthine bound structure) and human (cyan, PDB ID 1RCT, inosine bound structure) PNPs were used for the structural comparison [64]. The active site is located in the vicinity of the intersubunit interface, and a structurally equivalent Phe172 is contributed from the neighboring monomer. Water molecules at probable phosphate and ribose binding sites in the *Klac*PNP and the calf spleen PNP are shown as blue and orange spheres, respectively. The two conserved water molecules involved in the water-mediated interactions are encircled (red broken circle). Ligands and amino acids in the active site are shown in the stick representation. Oxygen, nitrogen, and sulfur atoms are shown in red, blue, and yellow colors, respectively. A sulfate ion occupies the potential phosphate binding site. Most of the residues forming the active site superpose well, except for variations in the turn connecting β1 and 3_10_ helix. For clarity, some residues, which are not a part of the active site, have been removed.

A catalytic site is formed in the vicinity of the intersubunit interface, where Phe172 from adjacent monomer participates in the formation of an active site pocket. Structural comparison of the hypoxanthine bound *Klac*PNP and the calf spleen PNP (PDB ID 1VFN) [61] structures revealed that the phosphate binding site is occupied by three water molecules in both the structures. Similarly, the probable ribose sugar binding site in the *Klac*PNP and the calf spleen PNP is occupied by four water molecules. Two water molecules, hydrogen-bonded to 6-O and 3-N of hypoxanthine, are involved in the water-mediated interactions in *Klac*PNP. These water molecules are conserved in the calf-spleen structure as well (Fig 6). The phosphate-binding site in the *Klac*PNP is primarily composed of Gly41, Ser42, Arg96, His98, Thr127, Asn128, Ala129, Met232, and Ser233, and the ribose sugar binding site is lined by Arg96, His98, Tyr100, Asn128, Ala129, Phe172, Phe213, Gly231, Met232, Ser233, His281, and Val284. The residues surrounding hypoxanthine in the *Klac*PNP are Ala129, Ala130, Gly131, Phe213, Glu214, Val230, Gly231, Met232, Thr255, Asn256, Cys258, and Val284. Cys258 in the *Klac*PNP is replaced by a structurally equivalent Val245 in the calf spleen (PDB ID 1VFN) [61] and human PNP (PDB ID 3PHB) [53], while residues lining the sugar binding site are identical.

### Hypoxanthine is intrinsically bound to the active site of *Klac*PNP

**Fig 7:**
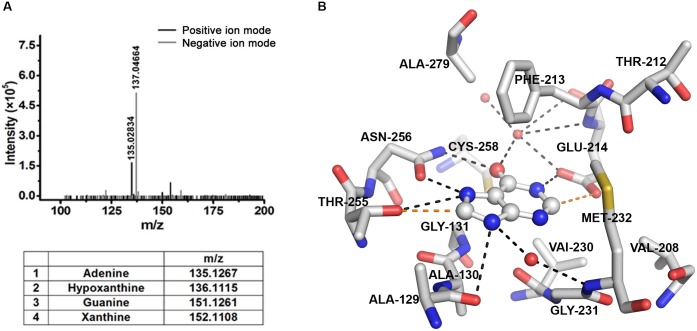
Active site architecture of *Klac*PNP with bound hypoxanthine in the active site. (A) ESI-MS analysis of intrinsically bound ligand (upper panel) and expected mass of the possible ligands (lower panel) is shown. Mass spectrometry analysis unambiguously suggests that hypoxanthine is bound to the active site. (B) Possible conventional hydrogen bond and weak C–H⋯O interactions are represented by broken black and broken red lines, respectively. The Phe213 side chain is oriented almost perpendicular to the substrate and mediates the π-π interactions. Hypoxanthine, amino acids, and water molecules are represented as ball and stick, sticks, and red spheres, respectively. The atoms are colored according to the following color code: carbon, grey; nitrogen, blue; oxygen, red.

Clear planar electron density at the active site in all monomers was observed in the crystal structure from the early rounds of model building and refinement cycles. No component present during protein purification or in the crystallization condition could be modeled satisfactorily in the said electron density. Subsequently, we built different substrates and end products of the reactions. Both hypoxanthine and adenine could be modeled well in the electron density. Since there is a mass difference of only 1 Da in both the ligands, we used ESI-MS analysis to confirm the identity of the bound ligand, as described in the methods section. Based on the observed mass, we could unambiguously identify the bound ligand as hypoxanthine (Fig 7A). The presence of hypoxanthine is logical, as *Klac*PNP utilizes inosine as a preferred substrate and produces hypoxanthine as one of the end products. The average B-factor of the hypoxanthine molecules (B-factor 26.6) and the surrounding residues (B-factor 22.94) is comparable, suggesting a near unit occupancy and, hence, high affinity of the ligand. Hypoxanthine remained bound to the protein throughout the protein purification steps and crystallization process, suggesting high affinity of hypoxanthine to *Klac*PNP. Hypoxanthine is stabilized by several interactions. In addition to five potential hydrogen bonds between protein and hypoxanthine, two indirect water-mediated interactions are also observed (Fig 7B). These water molecules are also conserved in close structural homologs and they participate in protein-ligand interactions [61]. Close inspection also revealed two weak C–H⋯O interactions (Fig 7B). The criteria of distance *d* <3.2 Å and angle *α* in the range 90° to 180° are used to calculate potential C–H⋯O interactions [65]. In addition to hydrogen bonds, π-π interactions mediated by the Phe213 side chain, lying almost perpendicular to the planar ring of the ligand, are also present (Fig 7B). There are several other non-bonded interactions as well. The presence of several stabilizing interactions may probably explain the high affinity of hypoxanthine to *Klac*PNP.

### *Klac*PNP is specific for 6-oxopurines

**Fig 8:**
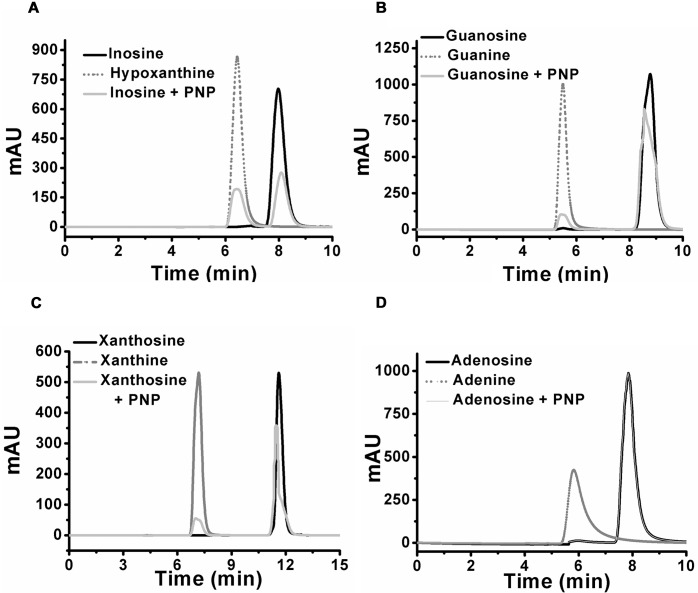
HPLC chromatogram of different substrates in the presence of *Klac*PNP. (A) Chromatogram showing the retention profile of inosine (solid black line) and hypoxanthine (dotted grey line) standards, and inosine + *Klac*PNP (solid grey line). (B) Chromatogram showing the retention profile for guanosine (solid grey line), guanine (dotted grey line), and guanosine + *Klac*PNP (solid black line). (C) Xanthosine (solid black line), xanthine (dotted grey line), and xanthosine + *Klac*PNP (solid grey line). (D) Adenosine (solid black line), adenine (dotted grey line), and adenosine + *Klac*PNP (solid black line). Adenosine with *Klac*PNP enzyme reaction showed no consumption of adenosine.

Intrinsically bound hypoxanthine observed in the crystal structure suggested inosine as a preferred substrate of *Klac*PNP. We determined kinetic parameters of *Klac*PNP with inosine and other 6-oxopurines. Our data suggest that *Klac*PNP accepted inosine as a preferred substrate with *K*_M_ (μM) and *k*_cat_ (s^-1^) of 21.0 ± 2.4 and 43.9 ± 3.1, respectively, with a catalytic efficiency of 2.0 × 10^6^. Kinetic parameters of *Klac*PNP, for inosine were comparable to those of human and *S*. *cerevisiae* PNP [43, 48]. Data presented in Table 2 suggest that *Klac*PNP accepts different 6-oxopurine substrates in the order inosine > guanosine > xanthosine. *Klac*PNP presented an approximately 25-fold lower *K*_M_ for inosine in comparison with that of guanosine and xanthosine, while *k*_cat_/*K*_M_ was about 40- and 60-fold higher, respectively. These studies confirm that *Klac*PNP is an inosine phosphorylase, as it accepts inosine as the preferred substrate. The substrate specificity was further demonstrated by HPLC analysis (Fig 8). No significant activity towards adenosine was detected, even in the presence of mM range of substrate concentration. Kinetic parameters of *Klac*PNP with different substrates are presented in Table 2.

**Table 2:** Kinetic parameters of *Klac*PNP, *Klac*PNP^N256D^, and *Klac*PNP^N256E^ with different substrates.

	Substrate	*K*_M_ (μM)	*k*_cat_ (s^-1^)	*k*_cat_/*K*_M_ (s^-1^M^-1^)
*Klac*PNP	Inosine	21.0 ± 2.4	43.9 ± 3.1	2.0 × 10^6^
	Guanosine	500 ± 29	29.7 ± 2.6	5.0 × 10^4^
	Xanthosine	555± 43	16.8 ± 0.6	3.0 × 10^4^
	Adenosine	ND	ND	ND
*Klac*PNP^N256D^	Inosine	222 ± 18	34.9 ± 1.2	1.6 × 10^5^
	Guanosine	842 ± 49	10.4 ± 0.4	1.2 × 10^4^
	Xanthosine	536 ± 40	23.3 ± 2.0	4.3 × 10^4^
	Adenosine	10.3 ± 1.6	22.8 ± 1.2	2.2 × 10^6^
*Klac*PNP^N256E^	Inosine	144 ± 5	25.1 ± 1.7	1.7 × 10^5^
	Guanosine	ND	ND	ND
	Xanthosine	ND	ND	ND
	Adenosine	ND	ND	ND

ND = not detected

### *Klac*PNP^N256D^ accepts both 6-oxopurines and 6-aminopurines as substrates

Bacterial hexameric PNPs, with few exceptions, are known to show specificity towards both 6-oxopurines and 6-aminopurines [66, 67]. This broad substrate specificity has been attributed to the presence of a catalytically important Asp residue, instead of Asn residue. Asn243 in human PNP has been reported to act as hydrogen bond donor to O6 of nitrogen base of inosine, thus playing the catalytic role in phosphorylation of 6-oxopurines [45–49, 63]. Similarly, in the crystal structure of *Klac*PNP, the structurally equivalent Asn256 forms the hydrogen bond with O6 of hypoxanthine, which is an end-product of inosine phosphorolysis (Fig 7). Therefore, to broaden substrate specificity, based on structural information and comparative sequence analysis, we mutated the catalytic residue Asn256 to Asp256 to yield a *Klac*PNP^N256D^ mutant. *Klac*PNP^N256D^ had a *K*_M_ of 10.3 ± 1.6 (μM) and *k*_cat_ value of 22.8 ± 1.2 (s^-1^), and a catalytic efficiency of 2.2 × 10^6^ for adenosine. Thus, the kinetic parameters of *Klac*PNP^N256D^ for adenosine and *Klac*PNP for inosine are comparable. The mutant *Klac*PNP^N256D^ had a *K*_M_ of 222 ± 18, a *k*_cat_ of 34.9 ± 1.2, and a catalytic efficiency of 1.57 × 10^5^ for inosine. The kinetic parameters of *Klac*PNP^N256D^ for xanthosine and guanosine were comparable to those observed for *Klac*PNP. Data presented here suggest that *Klac*PNP^N256D^ has enhanced activity towards adenosine and can utilize 6-oxopurines and aminopurines as substrates in the order adenosine > inosine > xanthosine > guanosine, suggesting reversal of substrate specificity. Detailed kinetic parameters of *Klac*PNP^N256D^ are presented in Table 2.

### *Klac*PNP^N256E^ is highly specific for inosine

We also created another mutant, *Klac*PNP^N256E^ (Asn256 to Glu256), to test its effect on substrate specificity and catalytic efficiency. Interestingly, *Klac*PNP^N256E^ was highly specific for inosine and no significant activity towards adenosine, guanosine, and xanthosine was observed, even at mM range of substrate concentrations. *Klac*PNP^N256E^ showed approximately 7-fold higher *K*_M_ and 10-fold lower catalytic efficiency for inosine, as compared to the *Klac*PNP (Table 2).

### Biochemical properties of *Klac*PNP and its point variants *Klac*PNP^N256D^ and *Klac*PNP^N256E^

**Fig 9:**
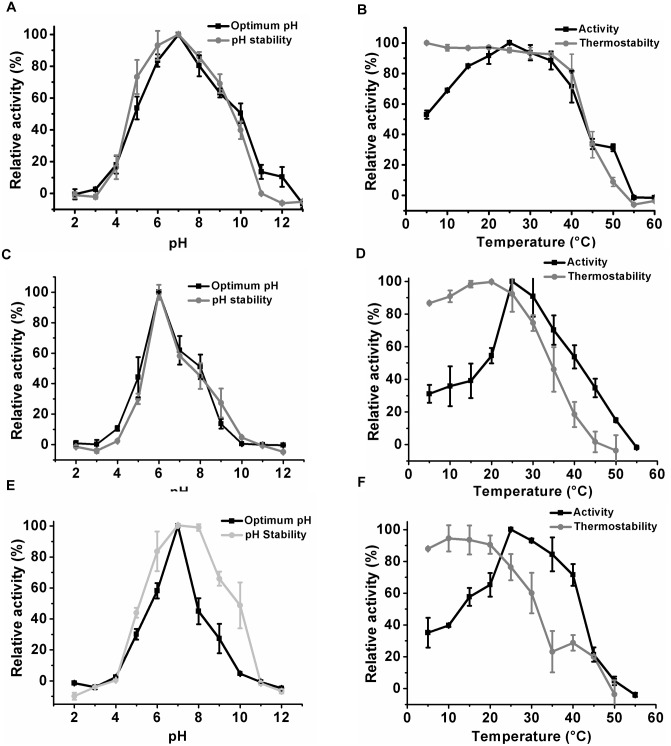
Biochemical characterization of *Klac*PNP, *Klac*PNP^N256D^, and *Klac*PNP^N256E^. (A) Effect of pH on the activity and stability of *Klac*PNP. (B) Effect of temperature on the activity and thermostability of *Klac*PNP. (C) Effect of pH on the activity and stability of *Klac*PNP^N256D^. (D) Effect of temperature on the activity and thermostability of *Klac*PNP^N256D^. (E) Effect of pH on the activity and stability of *Klac*PNP^N256E^. (F) Effect of temperature on the activity and thermostability of *Klac*PNP^N256E^.

A series of buffers at different pH values were used to test the activity of *Klac*PNP, *Klac*PNP^N256D^, and *Klac*PNP^N256E^ enzymes. Our data suggest that *Klac*PNP can function across a wide pH range of 5–10, with optimum activity observed at pH 7.0 (Fig 9A). The optimum pH of *Klac*PNP is comparable to the optimum pH values reported for human, calf spleen, and *S*. *cerevisiae* PNPs: 7.0, 7.0, and 7.5, respectively [43, 48, 68]. Higher molecular weight hexameric PNPs of prokaryotic origin are in general more thermostable, as compared to the low molecular weight trimeric PNPs [41]. *Klac*PNP also retained about 30% activity at lower and higher temperature limits of 5°C and 45°C, respectively, and the optimal activity was observed at 25°C (Fig 9B). The optimum temperature of *Klac*PNP was 25°C, which is similar to that reported for other PNPs of human, *E*. *coli*, and calf spleen origin [40, 61, 69], whereas *S*. *cerevisiae* works optimally at 30°C [43]. *Klac*PNP retained about 30% activity upon incubation at 45°C for two h, suggesting moderate thermal stability. Mutants *Klac*PNP^N256D^ and *Klac*PNP^N256E^ were also active between a pH range of 5.0 to 8.0, with optimum pH values of 6.0 and 7.0, respectively (Fig 9C and 9E). Mutants *Klac*PNP^N256D^ and *Klac*PNP^N256E^ showed optimum activities at 25°C and about 50% of activity up to temperatures of 35°C and 30°C, respectively (Fig 9D and 9F).

### *Klac*PNP and *Klac*PNP^N256D^ reduce purine content in a beer sample

**Fig 10:**
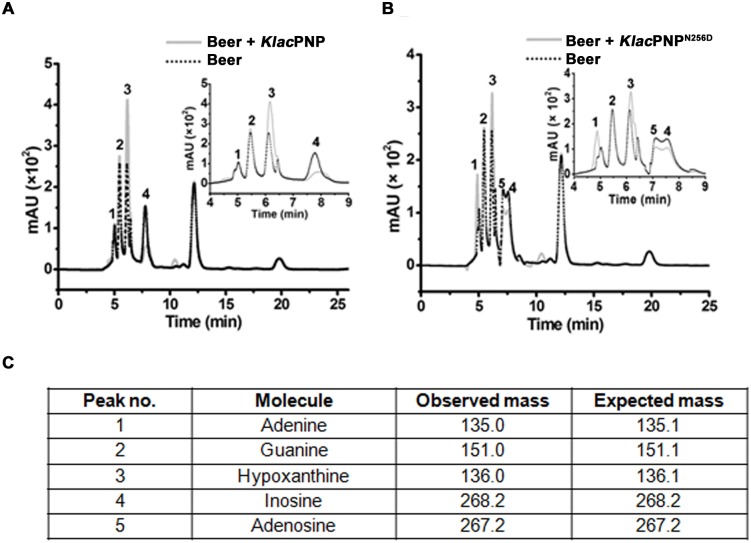
HPLC analysis of the beer sample before and after the enzyme treatment. (A) The chromatogram shows purine content in the beer sample before (dotted back line) and after *Klac*PNP enzymatic treatment (solid grey line). (B) The chromatogram shows purine content in the beer sample with added adenosine (20 mg/L) before (dotted back line) and after *Klac*PNP^N256D^ mutant enzyme reaction (solid grey line). Beer sample treated with 0.8 U of *Klac*PNP and *Klac*PNP^N256D^ mutant. In the panel (A) there is a decrease in the intensity of the inosine peak (4) and an increase in the hypoxanthine peak intensity (3). In the panel (B), there was a decrease in the inosine (4) and adenosine (5) peak intensities and corresponding increase in the hypoxanthine (3) and adenine (1) peak intensities, suggesting the conversion of inosine and adenosine into hypoxanthine and adenine, respectively. (C) ESI-MS analysis performed for the peak fractions. Expected and observed molecular mass of adenine, guanine, hypoxanthine, inosine, and adenosine are shown in Table 3.

The HPLC protocol for estimation of the purine content in the sample was initially standardized by using pure purine compounds [70, 71]. The *Klac*PNP-treated samples showed a decrease in the relative peak intensities for the substrates *i*.*e*., inosine, guanosine, xanthosine, and an increase in the peak intensities for the corresponding products *i*.*e*., hypoxanthine, guanine, xanthine, respectively (Fig 8). Beer sample was not pre-treated before incubation with the enzymes. Both untreated and treated (beer sample treated with the recombinant *Klac*PNP) samples were examined by HPLC (Fig 10). These peaks were further subjected to ESI-MS to confirm the identity of the compounds. We quantified the purine content in the beer sample and observed an abundance of purines in the following order: hypoxanthine > guanine >inosine> adenine (Table 3). Post *Klac*PNP treatment, samples showed a significant decrease in the inosine peak and a corresponding increase in the hypoxanthine peak. Since adenosine was not detected in the beer sample, we supplemented a beer sample with 20 mg/L adenosine, and this supplemented sample was treated with *Klac*PNP^N256D^. We observed a significant decrease in both adenosine and inosine peak intensities and a corresponding increase in the intensities of the adenine and hypoxanthine peaks, respectively.

**Table 3:** Purine content in the beer sample before and after enzymatic treatments.

Sample	Adenine (μMol/L)	Guanine (μMol/L)	Hypoxanthine (μMol/L)	Inosine (μMol/L)	Adenosine (μMol/L)
**Beer**	42.0 ± 1.1	69.4 ± 1.1	81.4 ± 0.4	94.7 ± 1.1	ND
**Beer + *Klac*PNP**	42.8 ± 0.6	72.7 ± 1.5	138.5 ± 2.5	30.7 ± 1.3	ND
**Beer**	42.4 ± 0.6	68.3 ± 1.1	79.0 ± 1.6	187.0 ± 1.9	
**Beer + *Klac*PNP**^**N256D**^	68.5 ± 0.6	72.0 ± 2.3	114.2 ± 1.7	111.8 ± 2.4	

ND = not detected. Column 5 and 6 in the last two rows are merged as peaks for inosine and adenosine in the beer sample supplemented with adenosine were not resolved well.

## Discussion

Hyperuricemia and gout are increasing worldwide and are affecting 1 to 2% of the adult population [72–74]. Hyperuricemia is also associated with atrial stiffness, cardiovascular diseases, and hypertensive pregnancy disorders [75, 76], and can be controlled by medication and change in lifestyle and eating habits [30, 31, 77]. Majority of the popular and energy-rich food items contain moderate to high purine content [77]. Practically, it would be difficult to follow dietary restrictions eliminating a large variety of foods. Moreover, it has been reported that about 80% of patients were unwilling to change their food habits and alcohol consumption [37]. Therefore, there is a need to produce low purine content food by using alternate approaches. Kunze’s group in Germany is working on one such attractive approach to exploit enzymes to reduce purine content in the food [78]. This approach seems practical and promising, as patients may find it easy to switch to their favorite low purine content food, rather than restricting its consumption. Beer is among the high purine content alcoholic drinks, and its consumption is reportedly increasing in some countries [79, 80]. Beer consumption is associated with an increased risk of developing gout [32]. Thus, consumption of low purine content beer might reduce hyperuricemia in patients by keeping the blood uric acid levels under control.

In the present study, we characterized *Klac*PNP, one of the key enzymes involved in the purine degradation pathway in *K*. *lactis*. Hexameric forms of PNPs are more stable and are devoid of long loop regions [41]. The most distinguishing feature of *Klac*PNP is the presence of a long loop connecting β9 and α7 (Fig 5), which participates extensively in establishing intermolecular interactions. Out of 24 hydrogen bonds at the interface, this loop region contributes to an average of five hydrogen bonds and several non-bonded interactions. This loop may probably play an important role in stabilizing *Klac*PNP. High-affinity hypoxanthine, observed to be intrinsically bound to the active site, forms extensive interactions in the active site. In addition to seven conventional hydrogen bonds, we also observed two potential C–H⋯O interactions that have not been reported earlier in the ligand bound PNP structures. Although the occurrence of such weak non-classical hydrogen bond interactions and their role in the structural stability and molecular recognition is well documented, these interactions are not so commonly reported in the context of protein-ligand interactions. We successfully used the structural information presented here along with the existing literature to engineer broad substrate specificity in the *Klac*PNP, which can be potentially exploited for lowering purine contents in beer and food samples.

To the best of our knowledge, we could not find any reports on purine content analysis of beer samples available in the Indian market. Therefore, we first determined the purine content in a popular Indian beer sample (Table 3). In the present study, we observed higher inosine and hypoxanthine content in the Indian beer sample, as compared to those in the analyzed beer samples from Japan and UK [77]. However, we could not detect significant amounts of other nucleosides. In previous reports, varying amounts of nucleosides, including adenosine, were detected in the beer samples [71, 81]. Hence, an ideal enzyme for treating a variety of food or beverage samples should have broad substrate specificity. Adenosine is not a preferred substrate for *Klac*PNP. Thus, we created *Klac*PNP^N256D^ and *Klac*PNP^N256E^ mutants, in anticipation that these point variants may have broad substrate specificity. The substitution of a catalytic residue Asn (Asn256 in *Klac*PNP) to Asp has been reported to introduce broad substrate specificity [48, 49]. Human PNP^N243D^ mutant can catalyze adenosine with a 5000-fold increased catalytic rate and a 2-fold lower preference for inosine substrate [48]. Dessanti and his group created the reverse mutation in *Bacillus cereus* AdoP^D204N^ (adenosine phosphorylase specific for adenosine) and found that this mutant presents a 430-fold decrease in *k*_cat_ for adenosine and prefers inosine as a substrate [49]. Our study suggests that the engineered *Klac*PNP^N256D^ point variant accepted all nucleosides: adenosine, inosine, xanthosine, and guanosine as substrates. This point variant accepted adenosine with the higher affinity and catalytic efficiency. Interestingly, there was no significant change in the *k*_cat_ for inosine, but a 10-fold decrease in the *K*_M_ and catalytic efficiency (Table 2). Catalytic efficiencies for guanosine and xanthosine did not change significantly. *Klac*PNP^N256D^ catalyzed inosine, xanthosine, and adenosine substrates with higher efficiency as compared to human PNP and *B*. *cereus* AdoP (adenosine phosphorylase). We believe that engineered *Klac*PNP^N256D^ will be able to reduce all four nucleosides if present in foods and beverages, whereas *Klac*PNP^N256E^ became highly selective for inosine with a 7-fold increase in *K*_M_ and a 10-fold decrease in the catalytic efficiency, in comparison to *Klac*PNP. We were successful in engineering both broad and narrow substrate specificities in *Klac*PNP by mutating Asn256 to Asp256 and Glu256, respectively. We tested the ability of *Klac*PNP and *Klac*PNP^N256D^ in catalyzing one of the key steps involved in lowering purine content in beer. We did not test *Klac*PNP^N256E^ with a beer sample, as it is highly specific for inosine. The treatment of beer sample with the enzymes showed a significant conversion of inosine to hypoxanthine with *Klac*PNP, and inosine to hypoxanthine and adenosine to adenine, respectively, with *Klac*PNP^N256D^ (Table 3 and Fig 10). These experiments demonstrate the potential applications of *Klac*PNP and its engineered variant in performing one of the essential steps aimed at lowering the purine content in a beer sample.

Biochemical properties of *Klac*PNP such as activity across a wide range of pH 5–10, moderate thermostability, and ability to retain >65% activity when stored at about 8°C for over a month, make it a suitable candidate for applications in the food and beverage industries. Once the components of the target food/beverage are established, then a cocktail of enzymes (PNP, adenosine deaminase, guanine deaminase, xanthine dioxygenase, and urate oxidase) can be utilized to effectively lower the overall purine content. For industrial applications, these recombinant enzymes can be immobilized on columns or mixed with food/beverage sample directly. Currently, our group is exploring strategies to make low purine content food affordable. However, the cost-effectiveness of this method using purified recombinant enzymes remains to be tested. We believe that using a yeast strain that overexpresses engineered enzymes like *Klac*PNP^N256D^, in combination with other enzymes involved in purine degradation for beer production, could be an attractive and cost-effective approach for producing low purine content beer. Further studies are needed to exploit the potential of enzymes in producing low purine content food at an industrial scale. Our study is in line with the ongoing efforts to develop alternative approaches that can help reduce uric acid levels in the body to control a number of diseases associated with hyperuricemia.
